# A safety app to respond to dating violence for college women and their friends: the MyPlan study randomized controlled trial protocol

**DOI:** 10.1186/s12889-015-2191-6

**Published:** 2015-09-08

**Authors:** Nancy Glass, Amber Clough, James Case, Ginger Hanson, Jamie Barnes-Hoyt, Amy Waterbury, Jeanne Alhusen, Miriam Ehrensaft, Karen Trister Grace, Nancy Perrin

**Affiliations:** Johns Hopkins University School of Nursing, SON 439, 525 Wolfe St, Baltimore, 21205 MD USA; Kaiser Permanente Center for Health Research, Portland, OR USA; Duke University Department of Psychiatry, Durham, NC USA

## Abstract

**Background:**

Research demonstrates high rates of physical and sexual victimization of women by intimate partners on college campuses (Black et al. 2001). College women in abusive relationships must weigh complex factors (health, academics, economics, and social stigma) during critical decision-making regarding the relationship. Rather than access formal support systems (e.g., campus security, administrators, counselors), research indicates abused college women most often turn to informal networks; specifically friends (Perspect Psychiatr Care 41:162–171, 2005), who often lack the knowledge or resources to provide effective support (Nurs Res 54(4):235–242, 2005). Decision aids have been shown to assist with health-related decisions by improving knowledge, creating realistic expectations, and resolving decisional conflict (Cochrane Database Syst Rev 1:1–332, 2014).

**Methods/Design:**

This study is a randomized controlled trial testing the effectiveness of an interactive safety decision aid web-based and smartphone application (App) for abused college women and their friends. Three hundred female college students experiencing abuse and three hundred friends of female college students experiencing abuse will be recruited in Maryland and Oregon and randomized to either the intervention safety decision aid, accessible by website or smartphone App, or a usual safety planning control website/App. The intervention App allows users to enter information on: a) relationship health; b) safety priorities; and c) severity of violence/danger in relationship. The App uses this information to provide personalized safety planning information and resources. Self-reported outcome measures for abused college women on safety seeking behaviors, decisional conflict, IPV exposure and mental health will be collected at baseline, six, and 12-months post-baseline via the study App/website. Outcomes measured for friends are IPV awareness, confidence to intervene, supportive behaviors and decisional conflict. Protocols for safely recruiting, retaining and collecting data from abused women via web/App are discussed.

**Discussion:**

This trial may provide important information on the impact of an App and web-based safety planning tool on college women’s decisional conflict and safety behavior use when making difficult safety decisions. This study is the first, to our knowledge, to test an intervention that engages friends of abused college women. The trial may also inform researchers on the feasibility of safely conducting research with abused women using online recruitment and enrollment methods and collecting data via an App or website.

**Trial registration:**

Clinicaltrials.gov ID: NCT02236663

## Background

Intimate partner violence (IPV) is established as a widespread problem with important negative health and social outcomes for women across the lifespan [1, 3, 5–12]. IPV is defined as threatened, attempted, or completed physical or sexual violence or emotional abuse by a current or former intimate partner. IPV results in an estimated 1200 deaths and 2 million injuries among women annually in the US [7]. Physical health conditions and risk behaviors are significantly higher among women who have experienced IPV compared with those who have not [3, 8, 9]. Beyond the physical conditions associated with violence, such as injuries and chronic pain, research has consistently demonstrated a strong association between IPV and increased rates of depression, post-traumatic stress disorder (PTSD), substance abuse, and suicide [13–15]. Researchers find disturbingly high rates of physical and sexual victimization of women by intimate or ex-intimate partners on college campuses, indicating that college campuses constitute at-risk communities for women [3, 8, 9, 16]. However, few colleges and universities have prevention and intervention programs for their students, particularly evidence-based approaches tested in controlled trials [17]. While an estimated 8 % of men will experience violence from an intimate partner in their lifetime, women’s rates of injury (41.6 % vs. 20 %) and death are far greater than men’s [18], therefore, our safety intervention for survivors will focus on college women.

One of the most widely recommended interventions to prevent and respond to IPV is safety planning [19–24]. A woman’s safety decisions for herself, her friends, and family are not linear and may change over time and during the course of a relationship. The safety process takes time, information, and resources, and involves consideration of complex individual factors (e.g. feelings for partner, privacy) and social factors (loss of social and financial support) [25–30]. Our challenge is to help college women develop a personalized safety plan that considers these complex factors during critical decision-making regarding safety while in an abusive relationship, and when ending an abusive relationship.

Rather than accessing formal systems for help (e.g., campus security, administrators, counselors), research indicates abused college women most often turn to friends [2], who often lack the knowledge or resources to provide effective support [3]. In a study by Banyard and colleagues [31], 1241 undergraduate students reported feeling conflicted about how to respond to a friend’s disclosure of IPV. The majority (64 %) reported feeling helpful, however, 43 % also reported being upset by their friends’ problems and only 20 % felt they knew how to help. In focus groups with young college women, Amar & Alexy [2] found a lack of understanding of IPV (e.g., victim-blaming attitudes, blaming violent incidents on alcohol), discomfort, and uncertainty how to respond were commonly reported. Female college students who are sexual minorities may face even greater challenges; while they are more likely than heterosexual female students to disclose violence or other relationship issues to friends, they are also more likely to report friends were unhelpful [32–34]. In our own work on campuses, friends of women who experience IPV often lacked knowledge of resources available on or off campus and lacked the confidence to intervene [35]. Arming peers with information and skills to support young women in risky relationships may therefore be a critical aspect of IPV prevention in this population. The influence of informal support systems on IPV survivors is a growing area of literature [2, 31–34], but little research focuses on the actual perspectives of friends of survivors and the complex decision-making process they are faced with when supporting a friend.

Evidence suggests that clinical decision aids can support informed decision making for patients faced with difficult choices (e.g. end of life decisions, treatment course for chronic illness) [4]. Decision aids have been shown to effectively reduce decisional conflict by providing information about options and risks involved and clarifying patients' personal priorities [4] Our team has developed MyPlan, the first, to our knowledge, decision aid designed to assist abused college women and their concerned friends with safety decisions. This paper describes the study protocol our team will use for the effectiveness evaluation of MyPlan. This protocol is currently approved by the Johns Hopkins Medicine and Kaiser Permanente Center for Health Research Institutional Review Boards (IRB).

## Methods

### Objectives

Our overall goal is to develop evidence-based interventions for college-aged women that prevent dating violence and limit the long-term negative physical and mental health consequences of IPV. Our objective is to evaluate the effectiveness and dissemination of an interactive, personalized smartphone application (“App”) intervention and website, named MyPlan, for: 1) college women (age 18–24) who experience IPV and 2) friends (age 18–24) of women experiencing IPV. The MyPlan safety decision App allows the user to enter information on: a) relationship health; b) safety priorities; and c) severity of violence/danger in the relationship. The MyPlan App then provides the user with a personalized safety plan with links to resources. Findings from our previous research with an internet-based safety decision aid [36] and from previous testing of the MyPlan App Prototype [37] suggest this approach offers college women and friends a unique opportunity to privately consider safety priorities, informs users about danger in the relationship, and provides an ongoing, accessible resource for safety. Therefore, our interdisciplinary team is examining two aims over 3 years:

- Aim 1. Test the effectiveness of MyPlan, an interactive, personalized safety decision App, on abused university/college women’s decisional conflict, safety behaviors, and IPV experience compared to women randomized to the control group (a generic IPV resource App). We hypothesize women in the MyPlan safety decision App group will have reduced decisional conflict about safety priorities which will lead to increased safety-behaviors (including reduction in alcohol/drug use) and reduced IPV at six, and 12 months post-baseline in comparison to the control group.
- Aim 2. Test the effectiveness of MyPlan, an interactive, personalized safety decision App, on abused university/college women’s friends’ (male and female) awareness of IPV, decisional conflict, confidence to intervene, and support provided to friends, compared to friends randomized to the control group (a generic IPV resource App). We hypothesize that friends in the MyPlan safety decision aid App group will have increased awareness of IPV and reduced decisional conflict about safety priorities, which will lead to increased supportive behaviors and confidence to intervene in comparison to the control group.

### Recruitment

To achieve Aim 1, we will recruit 300 women (age 18–24 years, *n* = 150 in Maryland and *n* = 150 in Oregon), enrolled at least part-time in college, who self-report current or past 6-months experience of physical violence, sexual violence, psychological abuse, or stalking by a dating/intimate/ex-intimate partner. Participants will be randomized to the intervention (MyPlan App) or the control group (generic IPV resource App). Outcomes for survivors will be collected by self-report at baseline, 6, and 12-months during the App session using validated measures. To achieve Aim 2, we will recruit 300 students (male or female) ages 18–24 years old (*n* = 150 in Maryland and *n* = 150 in Oregon) who are: 1.) enrolled at least part-time in college and 2.) report having a female friend with current or past 6-months experience of physical violence, sexual violence, psychological abuse, or stalking by a dating/intimate/ex-intimate partner. Friends of survivors will be randomized to the intervention (MyPlan App friend component) or the control group (generic friend IPV resource App). Outcomes (awareness of IPV, decisional conflict, supportive behaviors and confidence to intervene) for friends will be collected by self-report at baseline, 6- and 12-months post App session using validated measures. Eligible participants will also need to have safe access to a computer or smartphone with internet (“safe” meaning access to a device an abusive partner doesn’t have access to). A safe email address that an abusive partner does not have access to is also required to receive study related information.

Participants will be recruited through multiple strategies proven successful in our recruitment and retention of diverse populations in previous studies. These include partnerships with campuses to post advertisements for the study on campus social media, student listservs, etc. and to hang recruitment flyers in locations where students may seek assistance (campus clinics, student affairs office, etc.) or gather for support (student center, sororities, counseling centers). Advertisements on websites such as Craigslist and on community agency websites or social media may also be utilized.

Survivors currently in an abusive relationship or experiencing ongoing abuse from an ex-partner are navigating their safety every day, and they know the communication methods that are safest for them. We know from our previous research with survivors that they often have barriers to contacting and communicating with IPV resources due to their partner’s monitoring of their phone, internet use, or email, but creatively find ways to safely communicate. Strategies include: using computers at a library or friend’s house, having an extra pre-paid phone that their partner doesn’t know about, opening a 2^nd^ email account that their partner doesn’t know about, etc. The abusive partner may also harass and monitor friends and family limiting their safe communication methods as well. The barriers for one survivor may not apply to another, as each situation is unique. Because the main risk associated with this study is retaliation from an abusive partner if participation is discovered, we want to give participants options for contacting and enrolling in the study in the way that they deem safest for them in order to not put participants at risk. Additionally, we do not want to bias our sample by only being able to recruit participants who can safely contact and enroll in the study via limited contact options. For this reason, we give participants multiple options for contacting and enrolling in the study in the way they deem safest for themselves. Potential participants have the option to visit a secure study website for information about the study, or inquire directly by contacting the study team by email or a toll-free study number.

### Eligibility check procedures on study website

A study website address will be advertised on recruitment materials. If a potential participant visits the study website, the website will follow standard domestic violence program website procedures to initially inform users about the potential for an abusive partner to monitor computer activity and asks users to access the website from a safe computer and provides a link to additional information on using the internet safely. Participants can then can review the study purpose online and answer a set of eligibility criteria questions to determine if they are eligible to participate. Links to community resources (e.g. crisis helplines) are provided for all users visiting the website. If a user answers the eligibility questions, the website will be programmed to alert them if they are eligible to participate and if not, direct them to relationship abuse resources. If the participant is eligible and interested in learning more, they will be asked to enter a safe email address or phone number into the website and will be asked to provide instructions on how to be safely contacted (e.g. only call in the mornings, not safe to leave a voicemail) by a Research Assistant (RA) to complete enrollment. Information about the risk associated with creating an email trail with the study staff will be given, and the participant can choose if this is safe or not for their specific circumstances. As emphasized previously, giving participants choices in how to enroll and participate in the study safely is essential to ensuring participants are not placed at risk. Both phone and email communication choices are detailed below.

If the participant is interested in learning more, indicated by entering safe contact information in the study website, an auto generated email will be sent from the study website to the RA with the participant contact information and the RA will begin attempts to contact the participant either by phone or email, depending on safest available communication options provided by the potential participant. Once the participant is reached, the RA will document how they heard about the study, as the different recruitment strategies are a rich source of information for planning dissemination approaches.

### Phone/email enrollment procedures

If a potential participant feels safer contacting the study staff by phone or email rather than via the study website, the RA will give the potential participant a brief description of the study and then assess for eligibility (verbally or via email, depending on safe available communication options provided by potential participant). In an effort to provide safe options for enrollment at every step, a variety of options for communication with the research team are provided to accommodate participant preferences. Some may not want to answer the eligibility questions online, for example, while others may prefer this option over talking with someone or emailing.

### Informed consent and enrollment

Once eligibility has been assessed, the RA will conduct informed consent with potential participants. In Maryland, we requested a waiver of signed consent from the IRB and will use an oral consent script to conduct informed consent in lieu of requiring a signed document linking the study participant to a relationship abuse study. In Oregon, a copy of written consent is provided for participants, but rather than requiring a written signature, participants indicate their consent via an electronic checkbox on their first visit to the App/website. As previously indicated, giving participants the choice of the safest method for communicating with the study staff and conducting informed consent is important for safety reasons. If communicating via phone, an informed consent script/form will be read to the participant and consent will be obtained on the phone and enrollment completed via phone. The informed consent script/form will detail the purpose of the study, the procedures, confidentiality, risk and benefits to participation, and the research team’s protocol for danger assessment, and suspected child or elder abuse or intent to harm self or others including suicidality during the course of the study. The RA will ask whether the participant has any questions, answer any such questions, and then ask if they consent to be in the study. Community resources (e.g. crisis helplines) will be offered even if the person decides not to participate or is not eligible.

If the participant indicates on the study website or to the RA via email that the phone is not the safest way to communicate and that they would like to continue by email, the RA will inform the participant that conducting informed consent via email means that the oral consent script containing detailed information about a relationship violence study with a request to indicate consent to participate, will create an additional linkage to the study, and to consider whether it is safer to do via phone. If the participant wants to continue by email, the RA will email the oral consent script in Maryland/written consent from in Oregon for the participants review, ask if there are any questions, and enroll via email. This will take place in several steps to ensure the participant has the opportunity to ask questions, and that the RA gets confirmation that the participant understands the consent information. The final step will be to ask if they agree to take part in the research study.

Once a participant consents to enroll, the RA will explain the purpose of collecting safe contact information for follow up surveys and will collect safe contact information. For this study population, safe contact methods will likely consist mainly of email or text contact, but may also include phone and mail. The study team will contact participants in the manner participants deem safest (see Retention section). At each contact point, participants will be asked if there have been any changes to their safe contact information.

### Accessing the App

Upon obtaining informed consent, collecting safety contact information, enrolling participants and entering their information into the secure study database, participants will be randomly assigned to intervention or control groups using a computerized stratified blocked randomization scheme. Randomization occurs automatically at the time of enrollment via a randomization algorithm. Participants will be randomized to complete either the MyPlan Intervention App or the Generic IVP Resource App (control). Stratification is based on state of residence (Maryland or Oregon), type of participant (friend/survivor), survivor having children (child/no child in home), and type of school (two year technical school, community college, four year state college or university, four year private college or university, or other). Blocked design will ensure that the proportion of women/friends in Maryland and Oregon assigned to one group or the other remains relatively constant throughout the study. Once the participant is randomized, an automated email from the RA will be sent to the participants’ safe email address containing the assigned (intervention or control) study App access instructions. The participant will have two options to access the App. The first option is to download the App to their smartphone from one of the major U.S. App stores, where it will be posted as a free App available to anyone with a smartphone. Once downloaded, the App is user and password-protected, like a bank App, preventing others not enrolled in the study (e.g., partners) from opening the App. The second access option is to use the smartphone’s or computer’s Internet browser to access a web-based version of the App via the study website. With either mode of access, users will enter an assigned username and password the first time the App is accessed. They will also be instructed to set a security PIN code as the primary means to access the app once logged in. Users can define any 4 digit code to be the PIN and if they need to reset the PIN, must first log out and then back in using their username and password. Participants will be instructed to remove the App once they have completed the session if unsafe to have on their device, though warned that it is not currently possible to delete apps from a purchase history. Data collected from participants is labeled as either study data or personal identifiers. In order to ensure the highest protection of privacy and security, all personal identifiers (only collected at study enrollment) are saved in a physically separate database in a HIPAA compliant network environment. Study data and personal identifiers are never combined during the course of the study. Detailed safety procedures have been developed and are explained in the Risks section.

### Baseline data collection

Participants access the study website or App to complete data collection. This has several advantages: the participant can complete the instruments whenever it is convenient, and the data are entered by the participant, eliminating at least two sources of error – misreading hand-written forms and data input error. Electronic data collection may reduce social desirability bias compared to face-to-face interviews [38, 39]. For ease and to keep participants engaged with the website and App, we use radio buttons (i.e., on-screen buttons that allow selection from a group of options) and clickable sliding bars to answer questions. Participants can contact the RA if they have technical problems with the website or App, questions or concerns about the study, or need help finding someone safe to talk to. If participants contact the RA for assistance with safety or distress, the RA will be trained to respond with compassion and will provide the participant with the National Dating Violence Hotline phone number and website. Participants who do not complete the baseline assessment within the first four days will be sent an automated reminder email. The RA will determine if the participants received the email/password, had any problems accessing the App, or had any follow-up questions. The RA will continue to follow-up with participants who have consented and have not completed the session using the safe contact information provided by the participant until: 1) the sessions are completed, 2) the participant indicates they have decided not to continue with the study, or 3) 6 weeks from the date of enrollment has passed. If the App session is not completed within following the 4 day auto-reminder email, the RA sends manual reminders via email (3 attempts) then text/phone (up to 5 attempts, up to 3 messages left) using safe contact information provided by the participant. The baseline window closes and attempts to contact cease 6 weeks after enrollment. If at 6 weeks the participant has not logged into the App/website or completed baseline measures, they will be dropped from the study.

### Outcomes

The primary outcomes for Aim 1 for survivors consist of:

- *Use of safety strategies*: The Safety Behavior Checklist adapted from Sullivan & Bybee [40] and Parker & colleagues [41] measures the range of strategies used by survivors to halt or escape violence. This instrument asks if each strategy has been used over the last 6 months and if the strategy was helpful in dealing with the abusive partner or abuse. The checklist includes use of formal services (e.g., Have you met/called/texted/instant messaged a professional that works on campus (e.g. a professor, campus administrator, campus staff at a women’s center or campus health clinic) about your partner hurting you (physically, sexually, or emotionally?, Did you get/try to get an order of protection (or restraining order) against your partner?) and informal safety steps (e.g., Have you developed a code/signal so others would know when you were in danger from your partner?, Have you identified a safe place on campus or in the community you could go if your partner became dangerous?). The percent of safety behaviors tried by survivor that she found helpful will be the primary outcome.
- *Decisional conflict immediately post intervention*: The decision process for survivors is measured with questions adapted from validated subscales of the Decisional Conflict Scale (DCS) [42, 43]. The DCS tests whether using this safety planning decision aid helps a woman to understand the advantages and disadvantages of safety planning options and to know her values related to them. The Decisional Conflict Scale discriminates between people who make decisions and those who delay making decisions. The DCS consists of twelve items (e.g., “I know the risks of taking steps to increase my safety in my relationship”, “I am clear about which benefits of the relationship are most important to me”, “I have enough support from others to make decisions about my safety”) with a 5-point likert response scale from ‘strongly agree’ to ‘strongly disagree’. The DCS provides a total score, which is a measure of the decision process, as well as scores for four subscales (feeling informed, certainty about decision, values clarity, and support), with higher scores on the DCS indicating a greater degree of decisional conflict (indicative of a poorer decision process) [39, 40]. Decisional conflict will be measured pre and post the first use of the decision aid.

The secondary outcomes for Aim 1 for survivors consist of:

(c) *IPV exposure*: The Composite Abuse Scale (CAS) is a 30-item validated comprehensive intimate partner violence screening measure with strong psychometric properties [48, 49]. The CAS measures 4 dimensions: Severe Combined Abuse, Emotional Abuse, Physical Abuse, and Harassment. This project will use an adapted response scale: “never, only once, several times or many times” and participants will be asked how often their partner did any of the items in the last 6 months. Example items include: “Told me I was stupid”, “Pushed, grabbed or shoved me”, “Followed me”, “Made me have sex when I didn’t want to”.
(d) *Mental health*: Depression is measured with the Center for Epidemiologic Studies Depression Scale, Revised (CESD-R) [44, 45]. A 20-item self-report measure screens for depressive symptoms in community samples. Items are rated based on frequency in the ‘past week or so’ from 0 (Rarely or none of the time—less than one day) to 4 (Nearly every day for two weeks). The CESD-R is a revision of the widely-used and validated CESD, with revisions reflecting DSM-IV criteria for depression. The revised CESD correlates highly with the original, demonstrates good to excellent face and construct validity and excellent internal consistency [44–46].
(e) *Drug and alcohol abuse*: A modified version of the Monitoring the Future Drug and Alcohol Questionnaire is used to measure alcohol and drug use. Participants are asked to self-report on how many occasions they have used alcohol in the last 30 days and in the last 6 months, as well as binge drinking behavior. Participants are also asked on how many occasions they have used drugs, broken down into 5 categories (marijuana, club drugs/hallucinogens, stimulants/narcotics, prescription drugs, other) in the last 30 days and last 6 months. [47]
(f) *Decisional conflict at 6 and 12 months*: In addition to measuring decisional conflict outcomes immediately post intervention, decisional conflict outcomes will be assessed at six and 12 months.

The primary outcomes for Aim 2 for friends of survivors consist of:

- *Intimate partner violence awareness*: Friends’ awareness of IPV is measured by a 3 scales: 1) Acceptance of General Dating Violence Scale [48], is a five item scale (e.g., There are times in a dating/intimate relationship when violence is okay, Someone who makes their partner jealous on purpose deserves to be hit) with responses on a 4 point scale from strongly disagree to strongly agree; 2) Knowledge of IPV myths is measured with 5 items developed by the research team (E.g. Leaving an abusive partner will stop the abuse, women can’t be abused by a female partner) with responses on a 4 point scale from strongly disagree to strongly agree; and 3) Perception of IPV Awareness and Seriousness are measured with 2 items developed by our team, adapted from items from Beeble et. al [49] (“In your opinion, how *common* are abusive dating/intimate relationships in college? very uncommon, uncommon, happens sometimes, common, very common”, “In your opinion, how *potentially serious* (damaging, harmful, or dangerous) are abusive relationships in college? no potential for danger or harm, generally don’t cause much harm, can have some harmful effects, have harmful effects but generally not serious, potentially very serious or dangerous”).
- *Confidence to intervene*: Friends’ efficacy to intervene is measured with adaptation of Self-efficacy to Deal with Violence Scale [50] used with adolescents, adapted to assess friends of survivors’ confidence to intervene. The 19-item measure assesses how confident a person feels that they can address relationship issues on a 4 point scale (“not at all confident, somewhat confident, confident, very confident”. Example items include: “How confident are you in your ability to… start a conversation with a friend regarding worrisome behaviors by her partner/ex-partner”, “…identify appropriate/useful resources/people on your campus to help a friend in an unhealthy or unsafe relationship?”.
- *Friends’ supportive behaviors*: Supportive behaviors are measured with a checklist adapted from multiple sources, including Sullivan & Bybee [40] and Parker & colleagues [41] and from the literature around family/friend responses as described by survivors and engagement behaviors from Latta and Goodman’s [51] model and Beeble et al.’s [49] Approaches to Helping measure. Items ask about what the participant has done to support a friend in an abusive relationship (e.g. let her stay with you for safety reasons, gave her information about relationship abuse resources such as a hotline) and if each behavior was perceived as helpful to the friend.
- *Decisional conflict immediately post intervention*: The decision process for friends is measured with questions adapted from validated subscales of the Decisional Conflict Scale (DCS) [42, 43] used for survivors for Aim 1. The 12 items test whether using this safety decision aid helps a friend to understand the advantages and disadvantages of options for helping their friend in an abusive relationship, and to know his/her values related to those options (e.g., “I know my options for helping my friend who is in an unhealthy relationship.”, “I am clear about which reasons for helping my friend are most important to me.”, “I have enough advice to make decisions about helping my friend who is in an unhealthy relationship.”

The secondary outcomes for Aim 2 for friends consist of:

- *Intimate partner violence awareness*: Friends’ awareness of IPV is measured by 3 scales: 1) Acceptance of General Dating Violence Scale [48], a five item scale (e.g., There are times in a dating/intimate relationship when violence is okay, Someone who makes their partner jealous on purpose deserves to be hit) with responses on a 4 point scale from strongly disagree to strongly agree; 2) Knowledge of IPV myths is measured with 5 items developed by the research team (E.g. Leaving an abusive partner will stop the abuse, women can’t be abused by a female partner) with responses on a 4 point scale from strongly disagree to strongly agree; and 3) Perception of IPV Awareness and Seriousness are measured with 2 items developed by our team, adapted from items from Beeble et. al [49] (“In your opinion, how *common* are abusive dating/intimate relationships in college? very uncommon, uncommon, happens sometimes, common, very common”, “In your opinion, how *potentially serious* (damaging, harmful, or dangerous) are abusive relationships in college? no potential for danger or harm, generally don’t cause much harm, can have some harmful effects, have harmful effects but generally not serious, potentially very serious or dangerous”).
- *Decisional conflict at 6 and 12 months*: In addition to measuring friends’ decisional conflict outcomes immediately post intervention, decisional conflict outcomes will be assessed at six and 12 months.

### Description of the MyPlan intervention App

After completing the measures in the App, the participant will then be directed to the next section of the App, the MyPlan intervention. For both friends and survivors, the intervention starts by listing information about common IPV myths, and then describes characteristics of healthy relationships. Next the participant is directed to the “My Safety” section, which is the Danger Assessment (DA) or the DA-Revised (for women abused by a female partner). The risk-assessment consists of self-report items on validated risk factors for repeat violence and lethal IPV, such as an increase in severity and frequency of violence, controlling behavior, jealousy, use of alcohol/drugs, and forced sex [52]. A weighted scoring algorithm provides an instant DA score (range 0–38) graphically with narrative on the level of danger [53, 54]. For example, the score is converted to the validated levels of danger such as: 1) variable danger (low risk, but situations change quickly, a score of less than 8); 2) increased danger (a score of 8–13); 3) severe danger (a score of 14–17); and 4) extreme danger (a score of 18 and above) [54]. The participant is provided with personalized messages about their danger based on their score. For friends, this section is titled “My Friend’s Safety” and the section includes the DA or DA-R, the friend will respond to the risk factors based on what they know/observe in their friends’ relationship. They will not likely be able to answer some of the questions, for example, “the partner forces her to have sex”, and therefore, the true risk is under-estimated. However, in our previous research using the DA to validate risk of severe violence/lethality, assessment of danger in the relationship by a friend/family member is consistent with reports by the abused woman herself [52]. Next, the App includes “My Priorities,” an interactive visual aid that allows users to set priorities for safety. A clickable “sliding bar” allows the woman to make pairwise comparisons of importance between priorities including privacy, feelings for partner, severity of violence, well-being of children and social support/status. These are then combined mathematically to generate priority weights. For friends, priorities include concern for friend’s safety, privacy, personal safety and social support/status. After setting priorities, participants receive a personalized graph with the results. Next, the woman/friend moves to “My Plan,” personalized with messages based on responses in the previous sections. For example, if a woman indicates in the “My Safety” section that her partner has stalked her, the personalized safety plan messages will include detailed information about stalking and resources available to increase her safety. For friends, the “My Plan” section includes personalized messages on how to privately discuss concerns and provide campus and off-campus resources that can be useful to support their friend. We will collect information on women’s/friend’s use of the App, including number of times the App is accessed, time spent using the App, and content accessed, for example.

### Description of the generic IPV resource App (Control App)

The control App is based on usual safety planning services college women and friends have access to on-campus and on the Internet through IPV websites. We will not use the DA or DA-R for risk assessment in the relationship as these are specific components of the safety decision App intervention, but we will assess risk factors for severe violence as identified on IPV websites (such as the Red Flag Campaign). The control group App will provide women and friends with brief safety planning information and IPV resources targeted to college students age 18–24 years. The control group App safety plan is not personalized to women’s/friends’ safety priorities and danger in the relationship. As detailed above, we will collect information on women’s/friend’s use of the control App, including number of times the App is accessed, time spent using the App, and content accessed.

### Follow-up sessions

Follow-up App sessions for both groups will be conducted at 6 and 12 months post-baseline. These time points are selected for several reasons, including findings from previous research that IPV frequency and severity often increases over the course of a violent relationship, making it important that participants have access to the App to input changes in risk factors for IPV and review their safety plan at multiple time points [20]. Further, follow-up contacts provide important opportunities for the RAs and study participants to reconnect related to the study and supports retention of participants that may have to relocate for safety. Participants will be encouraged by email, text and phone contact by the RA to access the password-protected App to complete the session and follow-up assessment. As noted above, for all outreach to participants, the RA will use safe contact information and preferred mode of communication provided by the participant. The post-baseline data collection will consist of the same questions collected at baseline but will focus on outcomes since the previous session. For example, participants will be asked about threatened or actual IPV since the previous session/assessment.

### Retention

Several methods used by the research team have proven valuable in retention of participants in longitudinal studies [55–58]. All retention activities will be adjusted to meet the safety needs of the participant, on an individualized basis. Retention strategies include:

- Asking participant at enrollment for several (at least two) names, phone numbers, e-mails of individuals (i.e., friends, roommate, resident assistant, campus staff, coach) who would know of their whereabouts and could be used as alternate safe contacts. We will also ask if texting and leaving a message for each phone number is safe. As part of each email, text or telephone contact the participant receives from the RA, she/he is provided with study contact information;
- Safely contacting participants throughout the study, this includes contacts in-between (2, 4, 8, 10 months) the scheduled post-baseline sessions to briefly assess for safety, confirm contact information, and ascertain any anticipated changes to contact information;
- Using a safe email address, an automated email is sent to the participant announcing a follow up App session (6 or 12-month) is due. The window opens and this email is sent 2 weeks before the 6 and 12-month follow up due date. If follow up at 6 or 12 month is not completed within 1 week of first email announcing follow up is due, an auto reminder email is triggered, followed by manual reminders from the RA via email (3 attempts) then text/phone (up to 5 attempts, up to 3 messages left). If unable to reach the participant via direct contact info, the RA will attempt to contact alternate contacts (family, friends listed up to 2 times each). The follow up window closes and reminders cease 6 weeks after sending the 6 or 12-month follow up due email;
- Providing compensation to each participant for his or her time and expertise. Compensation for baseline ($20), 6-month ($30) and 12-month ($50) is a total of $100 for an estimated 3 hours of time, an amount consistent with other studies;
- Use of a secure study database for organizing participant contact information and study schedule.

## Data analyses

Before any analyses are carried out, the data will be audited for quality and completeness, including missing data patterns, evaluation of distributions for outliers in quantitative data, and checking distributions of variables to ensure that they meet the assumptions of planned analyses. Chi-square tests for categorical variables and t-tests for continuous variables will be used to test for differences between the safety intervention and control groups on baseline variables that are potential confounding variables. These included relationship status (dichotomous: currently residing with the abuser or not); language (English or Spanish), baseline knowledge of safety options (continuous); clarity of safety preferences (continuous); perception of support (continuous); computer experience (continuous). Variables for which significant differences are found will be included as covariates in the analyses where appropriate. All analyses will use intent-to-treat principles.

### Intervention effectiveness

Residualized change regressions will be used to test for significant differences between the intervention/control groups in change in decisional conflict. Change on the Decisional Conflict subscales, e.g., values or priorities clarification from pre-to post-use at the first session will be the dependent variable. Group (intervention vs. control), baseline decisional conflict and potential confounding variables for which intervention and control groups differ at baseline will be included in the model as independent variables. Longitudinal multilevel models will be used to examine change over time in women’s safety behaviors (percent of safety behavior tried that a survivor found helpful), IPV (measured by the CAS) and substance use (measured by AUDIT and DAST). Time (e.g. baseline, 6 months, and 12 months) will be used as a level 1 predictor of the dependent variable. The level 1 model yields an intercept and slope parameter for each person. The intercept reflects the baseline value on the dependent variable and the slope reflects the rate of change across time on the dependent variable. Group will be used as a level 2 predictor of slope parameter. Finding the group is a significant predictor of the level 1 slope parameters means that individual change over time differs for intervention and control groups. Potential confounding variables for which intervention and control groups are significantly different at baseline will be entered as level 2 covariates predicting individual change.

The same set of analyses described above will be used to examine change in friend’s awareness of IPV, decisional conflict, safety behaviors and confidence to intervene.

### Mediation analyses

Additionally, change in decisional conflict will be tested as a mediator of the relationship between the intervention and safety behaviors at 12 months for both survivors and friends of survivors using a series of regressions. Sobel’s test will be used to estimate the mediation effect. The product coefficient Approach associated with the Sobel’s test has been found to be a powerful method for estimating indirect effects [59]. The mediation effect and associated boot-strapped 95 % confidence intervals will be estimated. Separate analyses will be conducted for the women and friends. A similar approach will be used to test safety behaviors at 12-months as a mediator of the relationship between the intervention and change from baseline to 12-months in IPV for the women and confidence to intervene for friends.

### Moderator analyses

In additional analyses for Aims 1 and 2, we will examine age, race/ethnicity and sexual minority status as possible moderators of the intervention effect on the main outcomes of safety behaviors and IPV. Included in these models will be a group (intervention vs. control) by moderator interaction term at the second level of the model as a predictor of the slope for time. This will allow us to test if the effect of the intervention varies by the level of the moderator.

### Dose response analyses

We will examine the relationship between the number of times or “dose” the App is used during the year and change from baseline to 12-months in the main outcomes of safety behaviors and IPV using multiple regression. We will control for baseline scores on the outcome measures in this analysis as the level of use of the App may depend on severity of violence. This analysis will examine if repeated use of the App results in greater improvement in the outcome scores.

### Statistical power

We based the power analysis on a repeated measures ANOVA, but propose to use multilevel modeling for the analyses which allows all cases with missing data to be included in the analyses. We do not have adequate estimates of the within and between variance to estimate power for the multilevel model directly. However, in general multilevel model approaches are more powerful than repeated measures ANOVA. We have powered the study at the .90 level rather than .80 as we want to have adequate sample sizes to conduct exploratory analyses of the moderators of the intervention effect if necessary. Since the primary purpose of the safety intervention is to increase safety-seeking behaviors, the power analysis is based on an effect size calculated from the results from a previous IPV intervention [36]. These investigators evaluated the effect on safety-seeking behaviors of a telephone intervention compared to usual safety services in Spanish-speaking and English-speaking women who obtained restraining orders. The effect size, f, was computed to be 0.58, and according to Cohen’s conventions, this represents a medium effect size. Using means obtained from the telephone intervention study, this effect size is equivalent to a mean change from baseline to follow-up of 14.4 % for the intervention group compared to a change of 10.4 % in the control group from baseline to 12-months. Assuming an autocorrelation of 0.3, 300 participants in the intervention and 300 participants in the usual safety services group provide 0.99 power to detect a significant group by time interaction at the 0.05 alpha level. Assuming an attrition rate of 20 % and the same parameters as above, the study will have 0.99 power to detect a significant effect of the intervention. Even in a scenario where the attrition rate would be 37.5 %, the study would still have adequate power greater than .90 to detect a significant group by time interaction. Our prior work in this area suggests that 20 % is a reasonable attrition rate and we have successfully used multiple strategies to reduce attrition as described above.

## Risks and safety

The research team is well aware that questioning women/friends about partner violence is a sensitive topic that raises important questions about safety [57, 60]. Potential risks to participants are loss of confidentiality, time involvement, fatigue, distress and embarrassment because of the nature of some of the questions, anxiety, depression, and potential retaliation from the abusive partner if they learn of her/his participation in the study. As compared to the risks of a person seeking services and communicating with domestic violence services in the community, and viewing domestic violence safety information on a website or App, the risks are no greater. These risks will be minimized by developing and training study staff on safety protocols. All participants will be informed about the potential risks in participating and measures to take to protect one’s self, including safety measure for smartphone/internet use. All participants will be notified that they can withdraw from the study at any time without penalty. While participants’ safety cannot be completely guaranteed, we feel that with our safety procedures there is minimal safety risk. Although the circumstances of abuse may be distressing for survivors and friends to discuss, traumatized persons generally find expression of feelings useful. The research plan was developed with deliberate attention toward minimizing the risk of harm to abused women and friends of abused women. Following these safety protocols in previous online research, no adverse events were reported.

### Protection against risk

Measures to protect the study participants  will follow the guidelines set forth by the Nursing Research Consortium on Violence and Abuse (NRCVA) [61]. We are extremely concerned about safety, and this concern underlies the rationale for our procedures and research. Confidentiality is considered primary to this study protocol. Implementing important safety procedures for the internet and handling study data will serve to protect participants from harm. During the study, participants may stop at any time. All participants and those not eligible for participation will be offered information on safety resources for IPV. No information will be given out to anyone outside the research team about whether a particular person participates in the study. The study will generally be referred to as a “College Safety Study”. Trained research staff will conduct all aspects of the study.

All research team members will be trained using the approved protocol. Research team members will complete online human subjects research training through their institution. Further, all research team members will receive and review documentation on policies and procedures for managing and reporting adverse events that might arise during the study. The study investigators, who have many years of experience in the field of IPV and intervention research, will train all research staff prior to their involvement in the study. The training will include sensitization to the experience of abused women/friends, recruitment and retention protocols and scripts, safety issues and response (including protocols for on-line and telephone contacts with participants as well as handling and reporting of suicidal ideation, child abuse or harm to others), maintaining confidentiality of all research participants, intervention protocols and fidelity to protocols as well as issues related to informed consent. All research staff members will be provided a training manual as a reference. Refresher training and assessment will occur at least annually in order to prevent drift from these research protocols for safety.

Risk will also be minimized by providing participants with choices to allow the participant to decide the safest way for them to communicate with the study. Other safety considerations will be to discuss with participants the potential for abusive partners to monitor online/phone activities and ensure participant has a safe email address and safe device they can access in order to participate, making sure participants are aware of the type of information we will send them via email, asking them how they prefer to be communicated with and if there are any safety instructions for each mode of communication, providing participants with information on methods of protecting their privacy on their devices (smartphone/computer), and storing the intervention related data which will be identified only by a study ID# on a separate database from the enrollment data which will include contact information. Potential participants will be provided with a description of the risks or discomforts and the anticipated possible benefits. Additionally, to minimize the linkages of a participant to the study, the study is requesting a waiver of signed consent and will conduct oral consent in lieu of requiring a document about a relationship violence study containing a signature.

### Safety mechanism for online data collection

A link, username, and password to the secure study App will be emailed to the safe email provided by participant. The App measures and intervention will not collect any protected health information (PHI) from the participant. There is risk that an abusive partner could uncover the trail of sites visited by the participant and discover the purpose of the study. This risk will be discussed in detail with all participants and all participants will be given instructions in the email with the survey web link for safe internet use including using a computer/phone their partner doesn’t have access to, setting strong password protection for all devices, accessing “private” browsing, the limitations of deleting Apps completely from purchase histories, and covering up tracks on web browsers’ history files.

### Safety mechanisms to ensure data privacy

All persons with access to data (PI, co-investigators, RAs, consultants) will rigorously follow procedures to ensure confidentiality of data. All data will be secured in a password-protected, secure server and database. Contact information (PHI) will be collected for the participants separately and maintained securely in the study database on our secure server. No persons other than the investigators and study RAs will have access to contact information. Contact information for participants will be destroyed following completion of the study. Only a single file will contain PHI linking subject-identifying information (names) with study ID code. These sensitive files containing identifying information will be encrypted. This additional security requires the end-user to have both access authority and a password for the encryption/encapsulation. All other study materials and files will only include the study ID code.

## Discussion

The study hast the potential to significantly advance the science of IPV prevention and response through multiple innovations. First, the MyPlan App is available both as a downloadable mobile version and a “web-based” App (i.e., accessible from any web browser). With 66 % of young adults (age 18–29) owning a smartphone and 97 % having access to the internet [62] this strategy ensures nearly universal access for our target population, as the “Apps ecosystem” includes not only mobile devices with internet access (smartphones, tablet computers, mp3 players) but also laptop and desktop computers [63]. Our team’s strategy of developing the App in both downloadable mobile and web-based versions is consistent with best practices for future information technology development [64] providing users with a choice for safely accessing MyPlan. Secondly, MyPlan is appropriate for college women in same-sex relationships, using a revised version of the Danger Assessment (DA) demonstrated to accurately predict violence in abusive female same-sex relationships by Glass et al. [65]. Including sexual minority women and providing a tool specific to their relationships and context is of critical importance for culturally competent safety interventions. Thirdly, to our knowledge, MyPlan is the first IPV intervention for college women that also engages friends, an important support system for this age group, in safety planning [17]. We believe this will be the first experimental study to evaluate the effectiveness of a safety intervention App with young women who experience IPV and with friends of young women experiencing IPV on university and college campuses. Addressing this paucity of research is critical because the stakes are high for young women and their friends faced with safety decisions and planning.

## Abbreviations

IPV: Intimate partner violence
App: Application
PTSD: Post traumatic stress disorder
DA: Danger assessment
RA: Research assistant
CESD-R: Center for epidemiologic studies depression scale, revised
CAS: Composite abuse scale
NRCVA: Nursing research consortium on violence and abuse
PHI: Protected health information
DC: Decisional conflict
IRB: Institutional review board
